# Anti-Obesity Effects and Underlying Mechanisms of Total Polyphenols from *Cydonia oblonga Miller* (Quince) in High-Fat Diet-Induced Obese Mice

**DOI:** 10.3390/molecules31152582

**Published:** 2026-07-24

**Authors:** Nulibiya Maihemuti, Yipaerguli Paerhati, Nawaz Khan, Kayisaier Abudurousuli, Dilihuma Dilimulati, Alhar Baishan, Alifeiye Aikebaier, Wenting Zhou

**Affiliations:** 1 Department of Pharmacology, School of Pharmacy, Xinjiang Medical University, Urumqi 830091, China; nurbiye24@stu.xjmu.edu.cn (N.M.); yipaerguli@stu.xjmu.edu.cn (Y.P.); nawaz_khan@stu.xjmu.edu.cn (N.K.); kayisaier@stu.xjmu.edu.cn (K.A.); dilihuma@stu.xjmu.edu.cn (D.D.); alhar@stu.xjmu.edu.cn (A.B.); alifeiye@stu.xjmu.edu.cn (A.A.); 2 Xinjiang Key Laboratory of Active Components and Drug Release Technology of Natural Medicines, Urumqi 830017, China; 3 Xinjiang Key Laboratory of Biopharmaceuticals and Medical Devices, Urumqi 830017, China; 4 Engineering Research Center of Xinjiang and Central Asian Medicine Resources, Ministry of Education, Urumqi 830017, China

**Keywords:** *Cydonia oblonga*, total polyphenols, obese mice, mechanism of action

## Abstract

Obesity is a global metabolic disease closely associated with dyslipidemia, insulin resistance, hepatic steatosis, and chronic oxidative stress. *Cydonia oblonga Miller* (COM, Quince) from Xinjiang Uygur Autonomous Region of China is a traditional medicinal and edible plant rich in polyphenols, flavonoids, polysaccharides, and other bioactive constituents. Our previous studies suggested that total polyphenols of *Cydonia oblonga Miller* (TPCOM) may exert promising anti-obesity effects. Objective: This study aimed to investigate the therapeutic effects of TPCOM on high-fat diet-induced obese C57BL/6 mice and to explore its underlying molecular mechanisms related to glycolipid metabolism. Methods: TPCOM was extracted and purified from Xinjiang *Cydonia oblonga* fruits, and its total polyphenol content was determined using the Folin–Ciocalteu method. C57 mice were randomly divided into normal diet, model, and TPCOM intervention groups. After 12 weeks of high-fat diet feeding and 6 weeks of TPCOM treatment, body weight was monitored continuously. Serum levels of triglyceride (TG), total cholesterol (TC), low-density lipoprotein cholesterol (LDL-C), high-density lipoprotein cholesterol (HDL-C), and total antioxidant capacity (T-AOC) were measured using commercial kits. Hepatic pathological changes were observed by hematoxylin–eosin (HE) staining. Bioinformatics analyses including GO and KEGG were performed to predict key targets and pathways related to lipid metabolism. The protein expression levels of PPARGC1A, FFAR1, KLF15, Adipolin, GLUT4, and phosphorylated p38 MAPK in liver tissues were detected by Western blotting. Results: TPCOM intervention significantly reduced body weight gain in obese mice in a dose-dependent manner. Serum biochemical assays showed that TPCOM decreased TC, TG, and LDL-C levels, increased HDL-C levels, and markedly enhanced total antioxidant capacity (T-AOC). Bioinformatics analysis suggested that PPARG and FFAR1 were highly expressed in liver tissue and may participate in glucose and lipid metabolism regulation. Western blot results confirmed that TPCOM significantly upregulated the expression of PPARGC1A, FFAR1, KLF15, Adipolin, GLUT4, and phosphorylated p38 MAPK in the liver of obese mice. Conclusions: TPCOM effectively ameliorates obesity, dyslipidemia, hepatic steatosis, and oxidative stress in high-fat diet-induced obese mice. The underlying mechanism may be related to the regulation of glycolipid metabolism, mitochondrial function, insulin sensitivity, and antioxidant signaling via activating the FFAR1–PPARG–p38 MAPK axis and downstream targets including PPARGC1A, KLF15, Adipolin, and GLUT4. This study provides a scientific basis and theoretical support for the development and application of TPCOM as a natural functional ingredient in the prevention and adjuvant treatment of obesity and related metabolic disorders.

## 1. Introduction

Obesity has become a severe global public health threat, closely linked to metabolic syndrome, type 2 diabetes and non-alcoholic fatty liver disease (NAFLD) [1]. WHO data from 2023 shows its prevalence has tripled over the past 50 years, with more than 2 billion overweight and 650 million obese individuals worldwide. Beyond simple energy imbalance, modern research has clarified that obesity pathogenesis arises from complex interactions between genetics, living environment, intestinal flora and neural regulatory circuits. High-fat diet (HFD) mouse models are widely accepted as reliable tools to recapitulate human obesity and associated metabolic dysfunction.

Natural plant polyphenols have attracted extensive attention as candidate metabolic regulators, owing to their inherent antioxidant, anti-inflammatory and lipid-modulating bioactivities [2]. Multiple published studies have validated that polyphenol extracts from food and medicinal plants relieve obesity-related metabolic disorders via multi-target pathways, including reshaping intestinal flora, restoring mitochondrial function and modulating core glycolipid signaling such as IRS/AKT-AMPK [3,4]. Representative polyphenol preparations can upregulate glucose transport proteins (GLUT4, IRS1/2) to accelerate lipolysis, reduce circulating lipid levels, and suppress the release of pro-inflammatory cytokines (TNF-α, IL-1β, IL-6) by blocking the AGE-triggered RAGE/MAPK/NF-κB cascade [4]. Despite these documented broad benefits, the anti-obesity potential of polyphenols extracted from *Cydonia oblonga Miller* (COM, Xinjiang quince) remains incompletely characterized, with no systematic in vivo mechanistic data available to date [5,6].

As a dual-purpose medicinal and edible specialty plant, the fruit and seeds of COM from Xinjiang are rich in polyphenolic compounds (mainly flavonoids and phenolic acids) and polysaccharides. Our prior cell-based work confirmed that crude COM extracts suppress 3T3-L1 preadipocyte differentiation, implying preliminary anti-adipogenic activity [7]. However, whether TPCOM exerts consistent therapeutic effects and its underlying molecular regulatory logic in intact obese animals has not been verified.

We previously optimized the AB-8 macroporous resin purification workflow for TPCOM and completed UPLC-MS/MS profiling of its dominant phenolic monomers; the total polyphenol content and compositional data listed in Table 1 and Table 2 are reproduced from our published phytochemical report [7]. This chemical characterization only provides raw material background, without any in vivo activity or mechanistic exploration. All other datasets in the present manuscript are original experimental outputs, encompassing network pharmacology prediction, molecular docking, HFD mouse phenotypic evaluation, serum biochemical testing, quantitative hepatic histopathology and Western blot validation of the FFAR1–PPARG–p38 MAPK regulatory cascade.

Distinct from our prior research that solely focused on chemical characterization of quince polyphenols, the present study highlights three original innovations. Firstly, we report the first in vivo experimental evidence demonstrating that TPCOM effectively mitigates HFD-triggered body weight gain, hyperlipidaemia and hepatic steatosis in mice, filling the absence of systematic animal validation in existing COM polyphenol studies [8]. Secondly, an integrated strategy combining in silico target prediction and hepatic tissue protein quantification was adopted to uncover a unique glycolipid homeostasis regulatory axis responsive to TPCOM, which clarifies its multi-targeted protective mechanism against glycolipid metabolic dysfunction [9]. Thirdly, our pharmacological findings provide modern empirical evidence supporting the dual edible and medicinal ethnopharmacological applications of Xinjiang Cydonia oblonga Miller, establishing solid experimental foundations for the translational exploitation of TPCOM as a natural functional ingredient for the prevention and intervention of obesity and metabolic syndrome [10].

## 2. Results

### 2.1. Preparation and Content Determination of TPCOM

**Figure 1:**
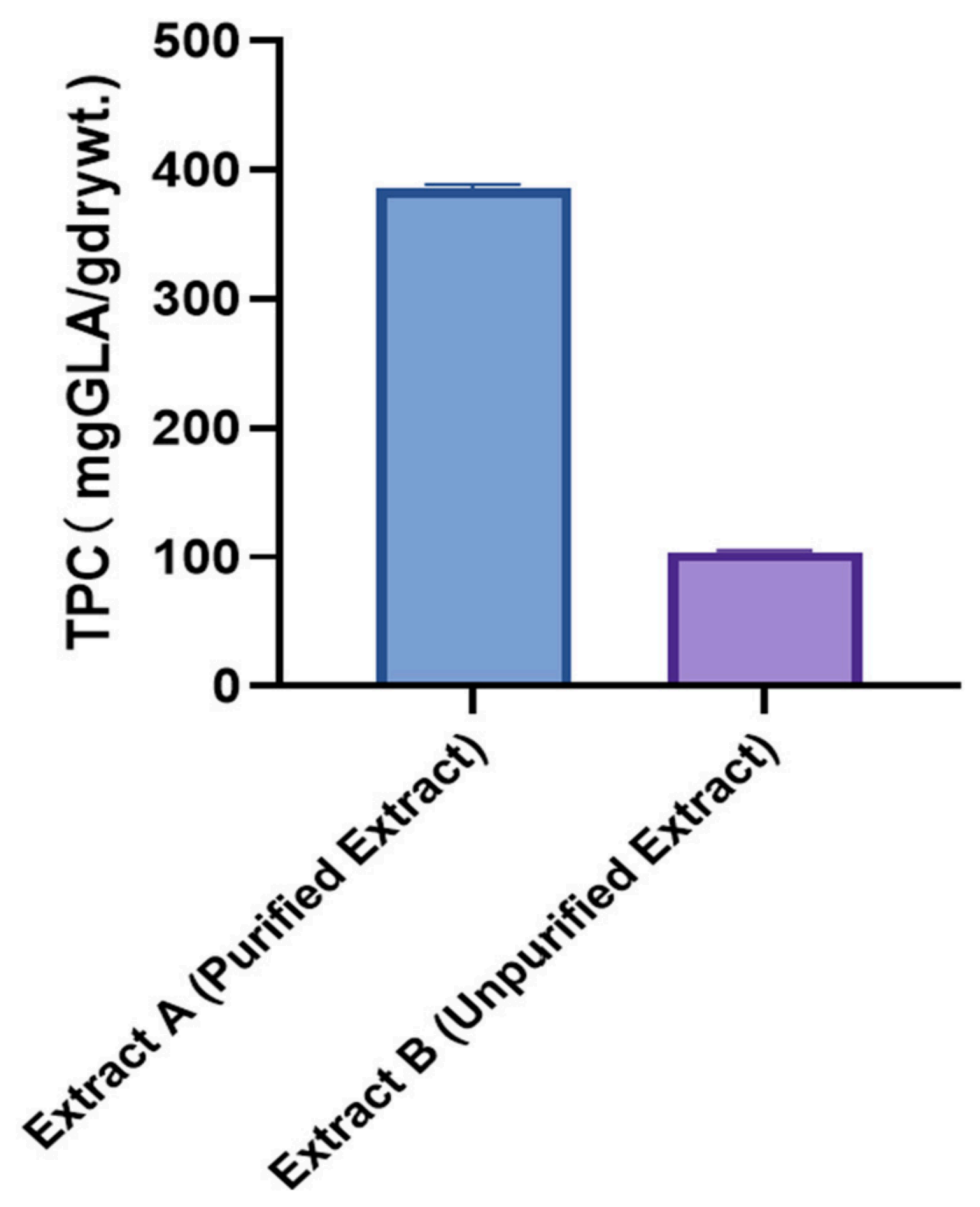
Total phenolic content (TPC, mg GAE/g dry weight) of purified and unpurified quince fruit extracts. Extract A: purified TPCOM; Extract B: crude unpurified extract. Data are presented as mean ± standard deviation (SD). AE: gallic acid equivalent.

The total phenolic content (TPC) of the purified and unpurified extracts was determined using the Folin–Ciocalteu method, and the results were expressed as mg gallic acid equivalents (GAE)/g dry weight. The purified extract contained 381.712 ± 0.529 mg GAE/g dry weight, while the unpurified extract contained 102.076 ± 0.607 mg GAE/g dry weight. As illustrated in Figure 1.

The chemical composition and quality control data of TPCOM have been fully reported in our previous publication. The chemical composition of the purified TPCOM used in this study was fully characterized and reported in our previous work using UPLC–MS/MS [7]. A total of 10 major polyphenolic compounds were unambiguously identified and quantified, including neochlorogenic acid, chlorogenic acid, cryptochlorogenic acid, caffeic acid, quercetin 3-rutinoside, quercetin 3-galactoside, quercetin 3-glucoside, kaempferol 3-rutinoside, kaempferol 3-glucoside, and isochlorogenic acid. Among them, cryptochlorogenic acid (242.44 ± 0.73 μg/mg), quercetin 3-rutinoside (186.33 ± 1.32 μg/mg), and chlorogenic acid (160.67 ± 2.51 μg/mg) were the most abundant components. The total quantified polyphenols reached 741.41 ± 9.04 μg/mg dry weight, with phenolic acids accounting for approximately 70% and flavonoids for 30%. The purified extract exhibited potent radical-scavenging activity, with EC_50_ values of 3.316 μg/mL (DPPH) and 36.38 μg/mL (ABTS). The same batch of purified TPCOM was used in the present anti-obesity study. Note that EC_50_ results are condition-dependent and cannot be directly compared with data from other laboratories without unified assay parameters.

As summarized in Table 1, 10 major polyphenolic compounds were identified in TPCOM, including neochlorogenic acid, chlorogenic acid, cryptochlorogenic acid, caffeic acid, quercetin 3-rutinoside, quercetin 3-galactoside, quercetin 3-glucoside, kaempferol 3-rutinoside, kaempferol 3-glucoside, and isochlorogenic acid. The quantitative results are shown in Table 2. Among these compounds, cryptochlorogenic acid, quercetin 3-rutinoside, and chlorogenic acid were the predominant constituents in the purified extract. These data indicate that TPCOM is a chemically characterized polyphenol-rich extract rather than an undefined crude preparation. The UPLC-MS/MS data summarized in Table 1 and Table 2 were obtained from our previously published study on quince fruit polyphenol extract prepared using the same extraction and purification procedure [7]. UPLC-MS/MS analysis was performed using an ACQUITY UPLC I-Class system coupled with a XEVO TQ-S triple quadrupole mass spectrometer (Waters Corp., Milford, MA, USA) equipped with an electrospray ionization (ESI) source.

All quantitative data of polyphenol monomers in this table are reproduced from our previously published research [7], which is only used to characterize the extract material for the subsequent independent pharmacological experiments in this work.

### 2.2. Network Pharmacology Analysis

#### 2.2.1. Screening of Active Ingredients and Target Information Analysis of TPCOM

To systematically explore the potential anti-obesity mechanism of *Cydonia oblonga*-derived extract, we first retrieved all documented phytochemical constituents of this plant from TCMSP and TCMIP databases to perform broad-spectrum network pharmacology analysis for preliminary target screening.

Subsequently, UPLC-MS/MS was used to characterize the actual chemical composition of purified TPCOM. Ten predominant polyphenolic components were identified as the main endogenous bioactive ingredients in our experimental samples, mainly including chlorogenic acid, caffeic acid, quercetin glycosides and kaempferol glycosides.

**Figure 2:**
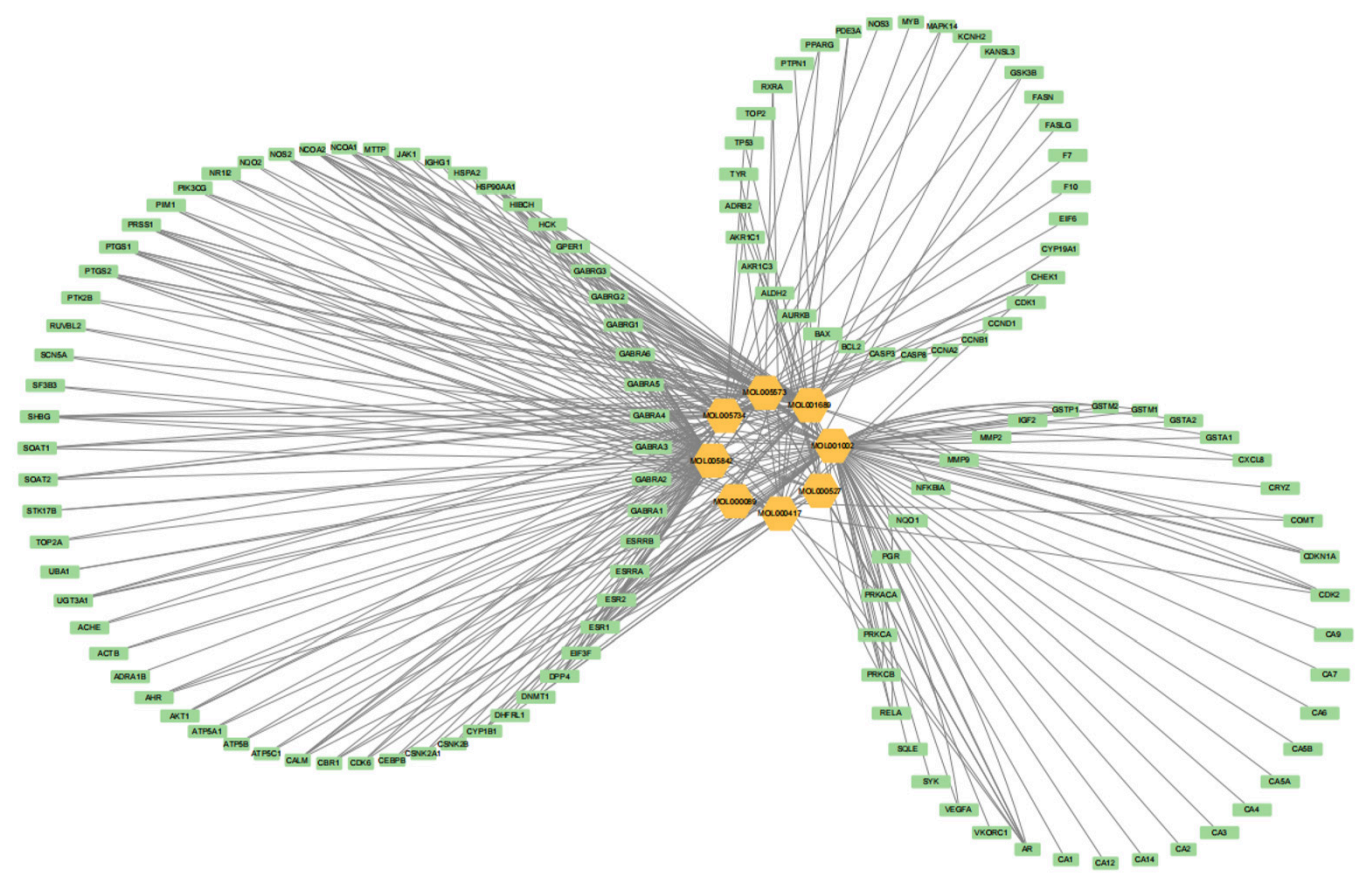
Protein–protein interaction networks.

On this basis, we further focused on these experimentally identified dominant components to extract their corresponding potential targets from the above databases. After eliminating duplicated genes, a total of 130 non-redundant putative targets were acquired (Table 3). The component-target interaction network was then constructed and visualized via Cytoscape 3.9.0 software (Figure 2). All core targets obtained from the above screening were further validated through subsequent in vivo and molecular experiments.

It should be clearly emphasized that network pharmacology and molecular docking in this study only serve as in silico predictive tools to generate preliminary mechanistic hypotheses, rather than direct functional biological evidence. All predicted target interactions and signaling cascades require subsequent in vivo protein expression detection for auxiliary verification, and cannot independently confirm the direct activation of any signaling pathway.

#### 2.2.2. Prediction of Potential Action Targets and Construction of an Active Ingredient Target Network of TCMSP Against Obesity

Obesity-related therapeutic targets were retrieved from GeneCards (Version 5.22, relevance score > 5) and OMIM databases. A total of 1559 obesity-associated genes were screened from GeneCards, and another 34 obesity-related genes were supplemented from OMIM. After merging and removing duplicate genes between the two databases, we finally obtained 1561 total obesity disease targets.

**Figure 3:**
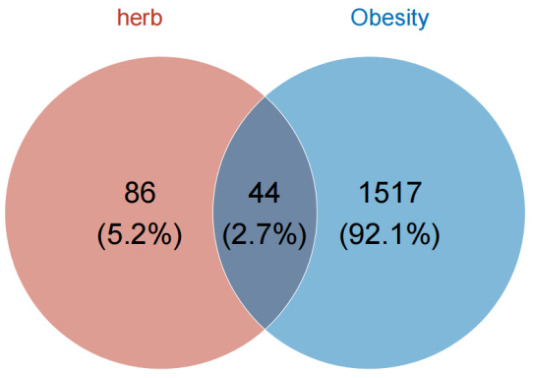
Drug–disease intersection targets.

We further conducted Venn intersection analysis between TPCOM candidate targets and the 1561 obesity targets. As shown in Figure 3, there were 44 shared therapeutic targets overlapping between TPCOM and obesity; 86 targets were uniquely owned by TPCOM, while the remaining 1517 genes belonged exclusively to the obesity target set.

#### 2.2.3. GO and KEGG Analysis

**Figure 4:**
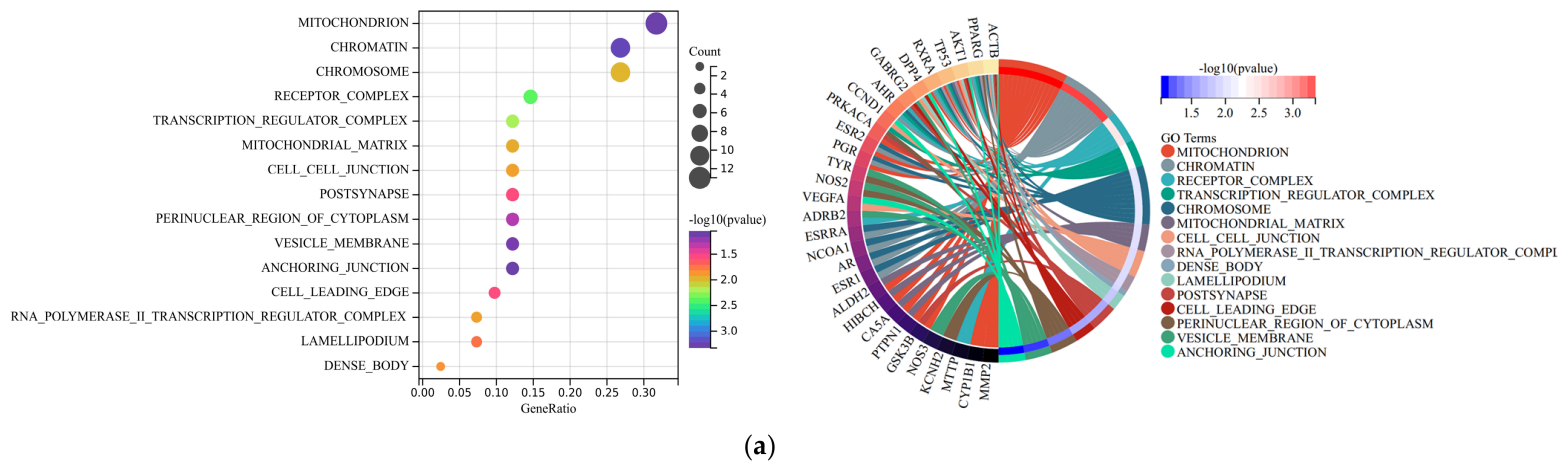

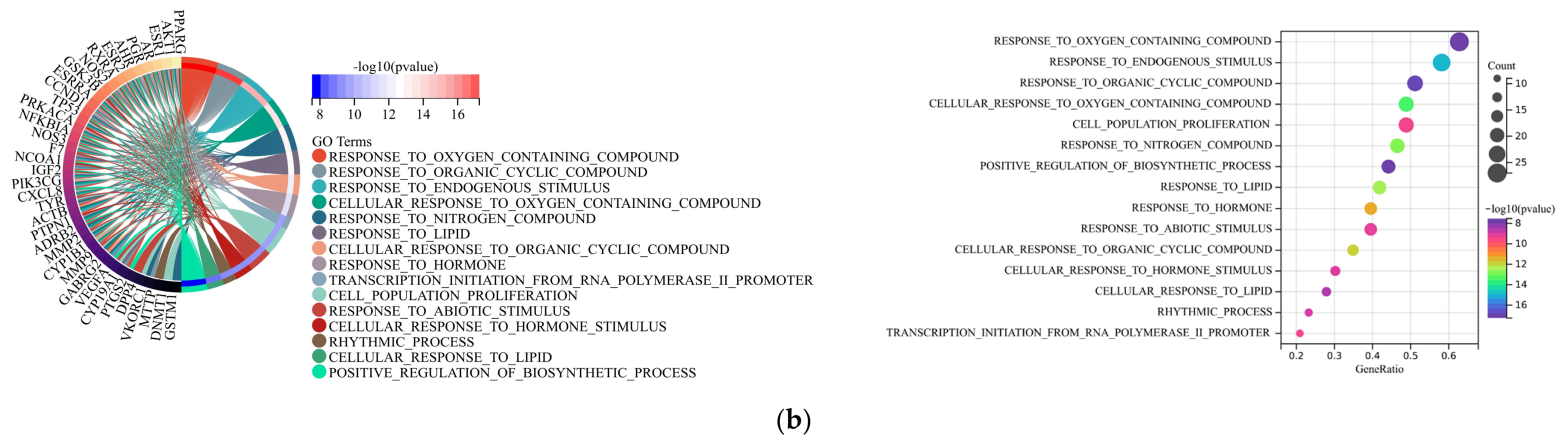
GO and KEGG enrichment analysis of overlapping target genes shared by TPCOM and obesity. (**a**) Bubble plot showing hub genes significantly enriched in mouse hepatic tissue; PPARG and FFAR1 were identified as core hub genes highly enriched in liver glycolipid metabolism-related pathways. (**b**) Bubble plots of top 20 significantly enriched GO biological process terms and KEGG signaling pathways. All enrichment analyses were performed using the DAVID database, and significantly enriched functional terms and pathways were screened with the statistical cutoff *p* < 0.01.

Gene Ontology (GO) and Kyoto Encyclopedia of Genes and Genomes (KEGG) pathway analyses were performed for TCMSP anti-obesity targets using the DAVID.database. The number of related targets was ranked in descending order. *p* < 0.01 was taken as the defining value of significantly enriched functions and pathways, and the top 20 pathways were selected. The results were visualized using R 4.0.4 software (Figure 4).

#### 2.2.4. Molecular Docking Verification

**Figure 5:**
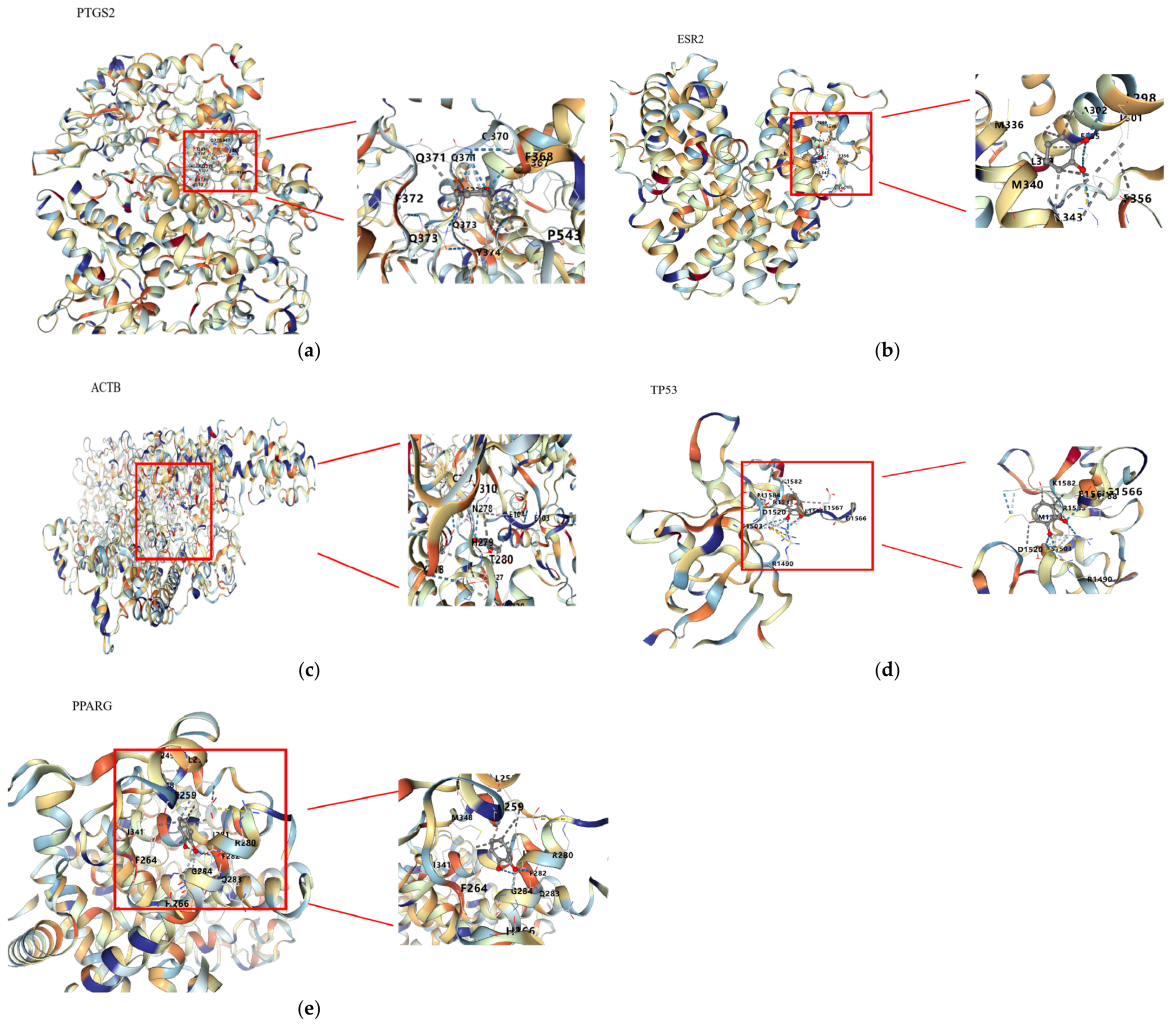
Representative molecular docking binding modes between dominant polyphenols of TPCOM and partial preliminary overlapping targets from TPCOM-obesity PPI network. (**a**) ESR2; (**b**) ACTB; (**c**) TP53; (**d**) PPARG; (**e**) FFAR1. Red boxes mark the ligand-binding pocket of polyphenol compounds inside each target protein. The binding free energy of ligand–protein complexes ranged from −7.2 to −9.6 kcal/mol.

Two distinct sets of target proteins were analyzed via molecular docking in this study. The first set consisted of representative overlapping targets (ESR2, ACTB, TP53, PPARG, FFAR1) derived from the TPCOM-obesity PPI network, whose docking binding modes are visualized in Figure 5. The second set contained five core glycolipid-regulating hub proteins (PPARG, FFAR1, p38 MAPK, PPARGC1A, GLUT4) with critical roles in lipid and glucose homeostasis; their detailed docking parameters are listed in Table 4, and these five proteins were further verified by hepatic Western blot in vivo.

As shown in Figure 5, the dominant polyphenols, including chlorogenic acid, cryptochlorogenic acid and quercetin 3-rutinoside, could form stable hydrogen bonds and hydrophobic contacts with amino acid residues inside the binding pockets of target proteins. The optimal binding free energies of ligand–protein complexes ranged from −7.2 to −9.6 kcal/mol, indicating favorable static binding affinity under simulated computational conditions.

Notably, these in silico binding data only serve as predictive auxiliary evidence. Satisfactory docking affinity cannot verify whether TPCOM can bind and activate these target proteins in hepatocytes, nor confirm the downstream cascade signal transduction of the FFAR1–PPARG–p38 MAPK axis. Consistent with our restrictive statement in Materials and Methods, all docking results only provide hypothetical interaction clues, and the actual regulatory effect of TPCOM on these proteins was validated by subsequent in vivo Western blot detection of hepatic protein expression [11].

### 2.3. TPCOM Reduced Body Weight Gain and Visceral Lipid Accumulation in HFD-Induced Obese Mice

**Figure 6:**
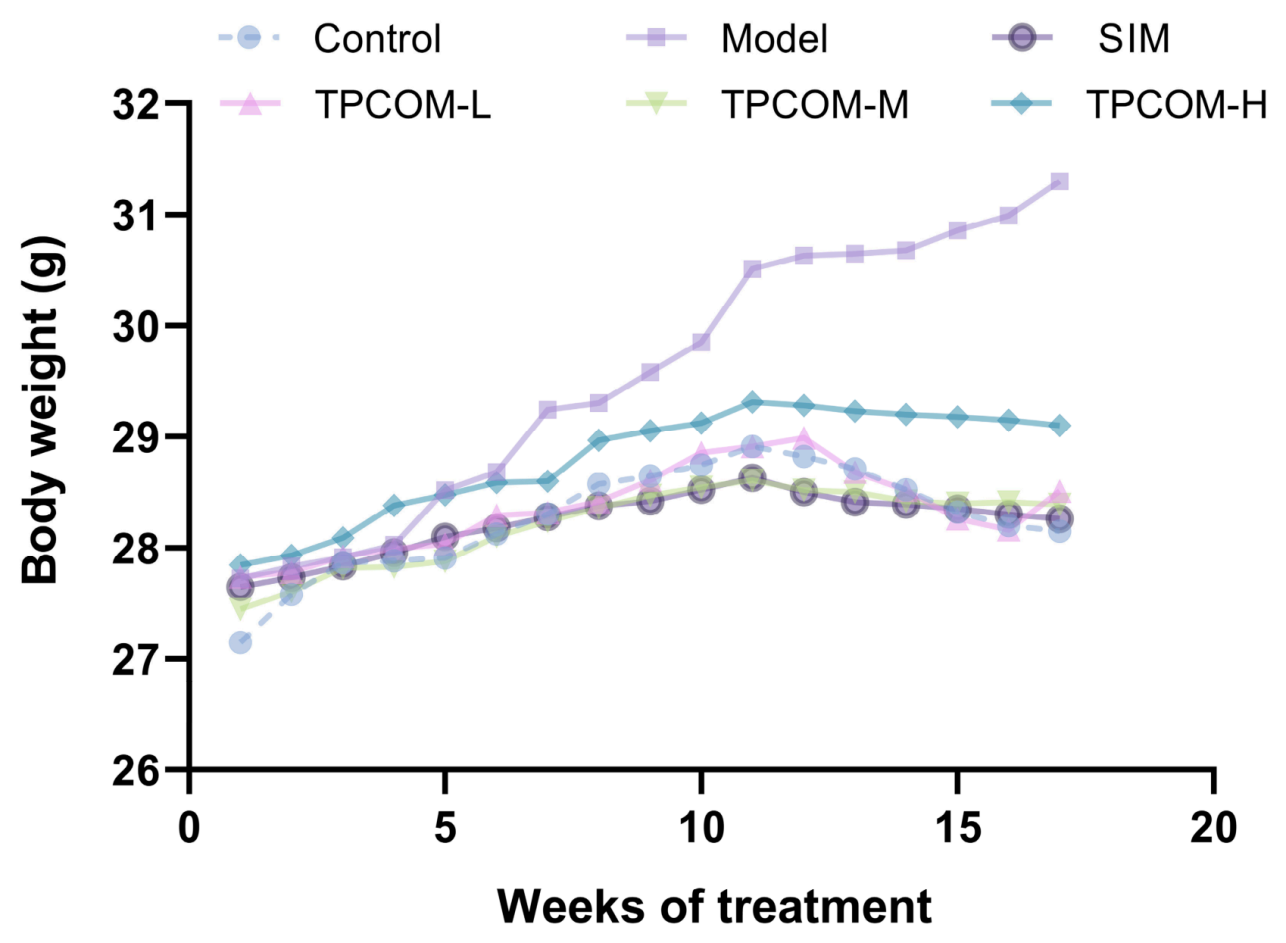
Dynamic body weight changes of mice during 6 weeks of intervention (n = 10 mice per group).

The dynamic body weight curves of six groups during the 6-week intervention are shown in Figure 6. The Control group maintained steady, mild weight growth within the normal physiological range, which was attributed to balanced lipid anabolism and catabolism under standard chow intake, without excessive lipid storage in adipose tissue and liver. In contrast, mice in the Model group displayed continuous rapid weight increase throughout the experiment, accompanied by greasy fur and decreased spontaneous activity, which confirmed the successful construction of a diet-induced obesity model [12].

**Figure 7:**
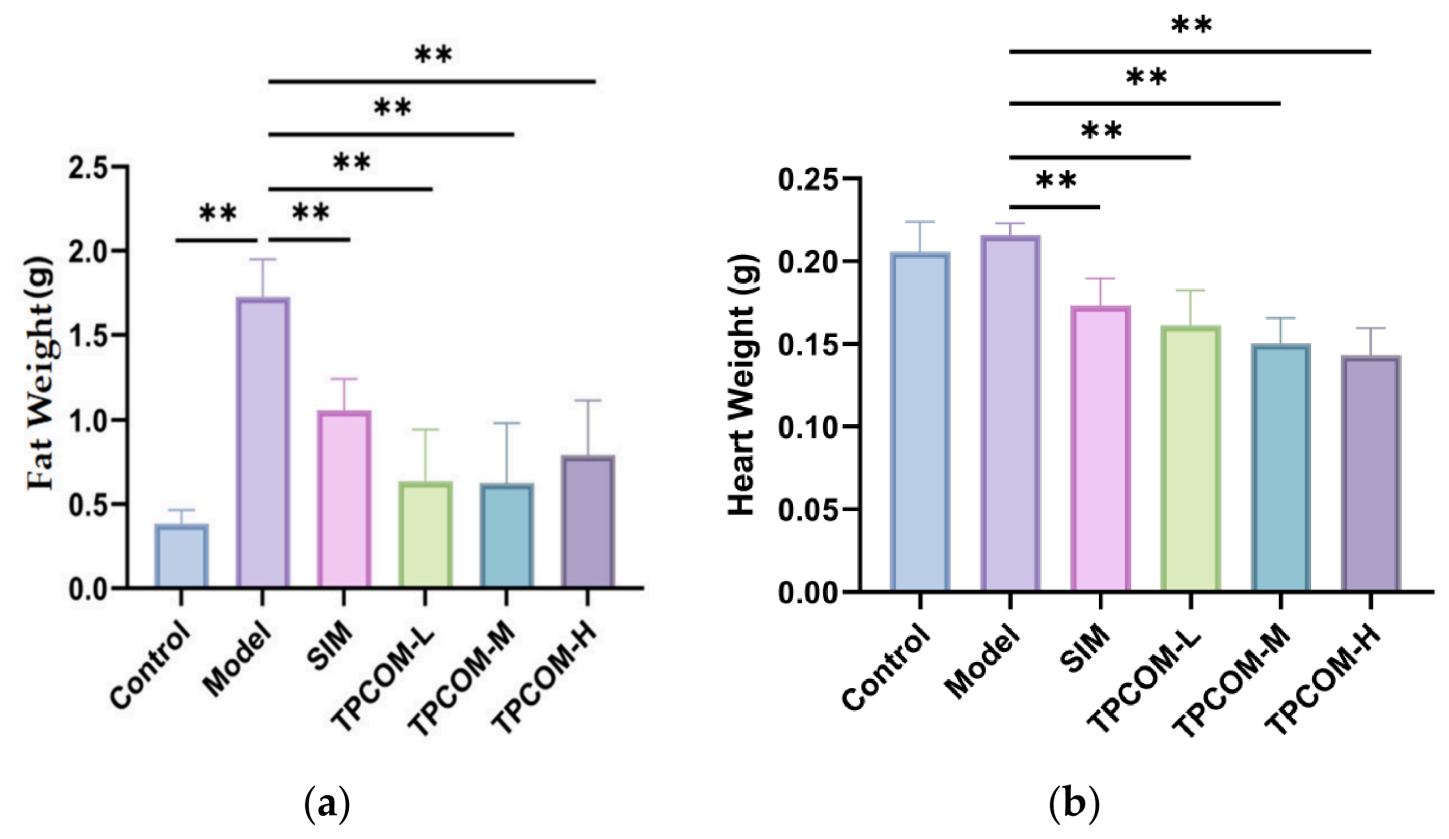

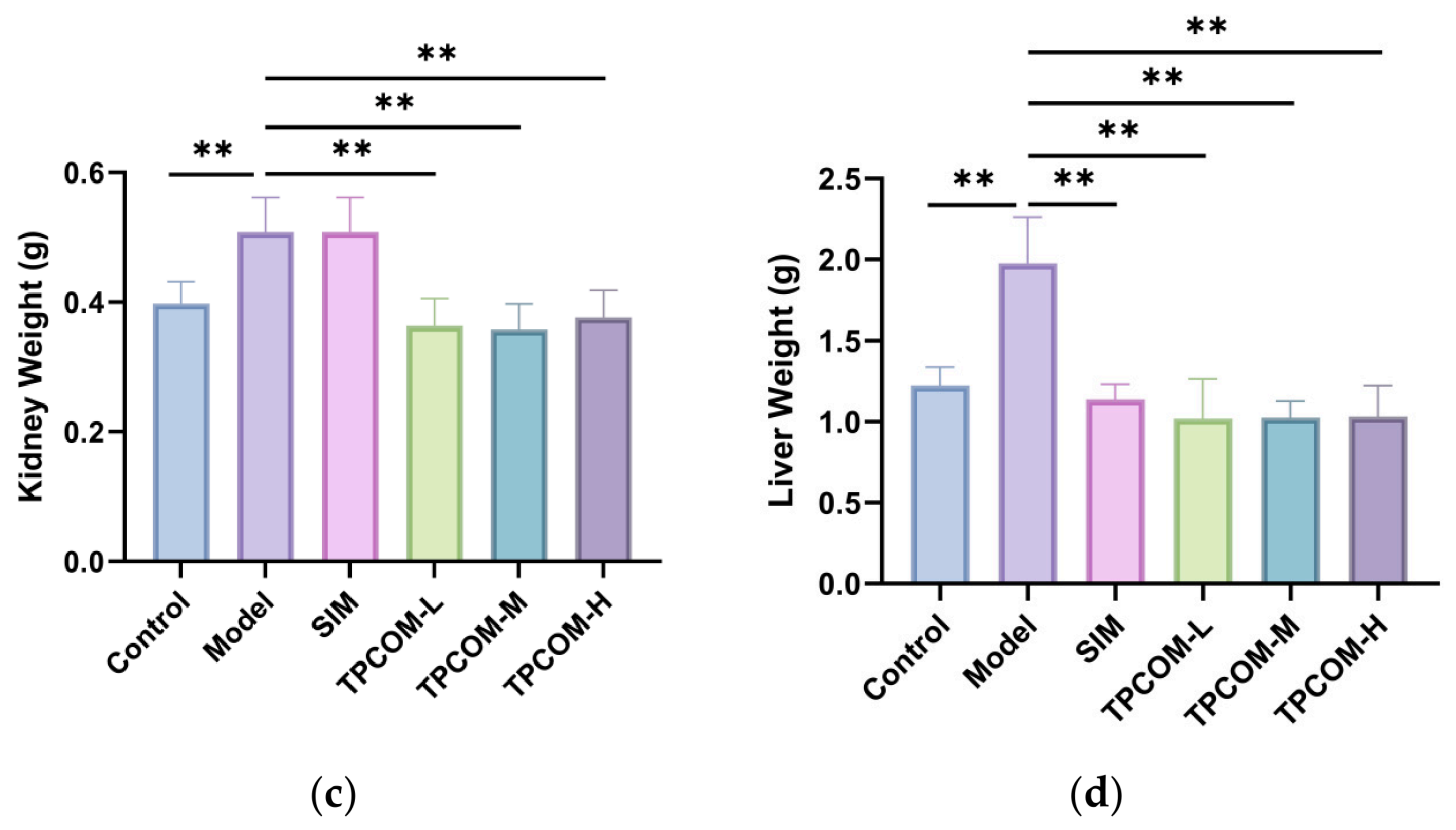
Wet weight statistics of visceral fat (**a**), heart (**b**), kidney (**c**) and liver (**d**) tissues (n = 10 mice per group). ** *p* < 0.01 vs. Model group.

After TPCOM intervention, the inhibition of excessive weight gain showed obvious dose-dependent characteristics. The TPCOM-L group presented a slightly slowed weight growth rate; the TPCOM-M group exhibited significantly suppressed weight elevation; The weight curve of the TPCOM-H group was similar to that of the SIM positive control group and far lower than that of the Model group at the end of treatment. No significant inter-group difference in food intake was observed during the whole intervention period, indicating that the weight-lowering effect of TPCOM was not caused by appetite suppression [13]. Organ weight statistics in Figure 7 showed that visceral fat and liver wet weight of Model mice were significantly higher than the Control group (## *p* < 0.01), which reflected severe visceral fat hypertrophy and HFD-triggered hepatic lipid deposition. The visceral fat and liver weights of mice gradually decreased with the increase of dosage, and the TPCOM-H group achieved obvious recovery (** *p* < 0.01). No statistical difference in heart and kidney weight was detected among all cohorts, which proved that TPCOM exerted no observable toxic effect on cardiac and renal tissues within the tested dosage range [14].

### 2.4. TPCOM Improved Serum Lipid Disorder and Elevated Systemic Total Antioxidant Capacity

**Figure 8:**
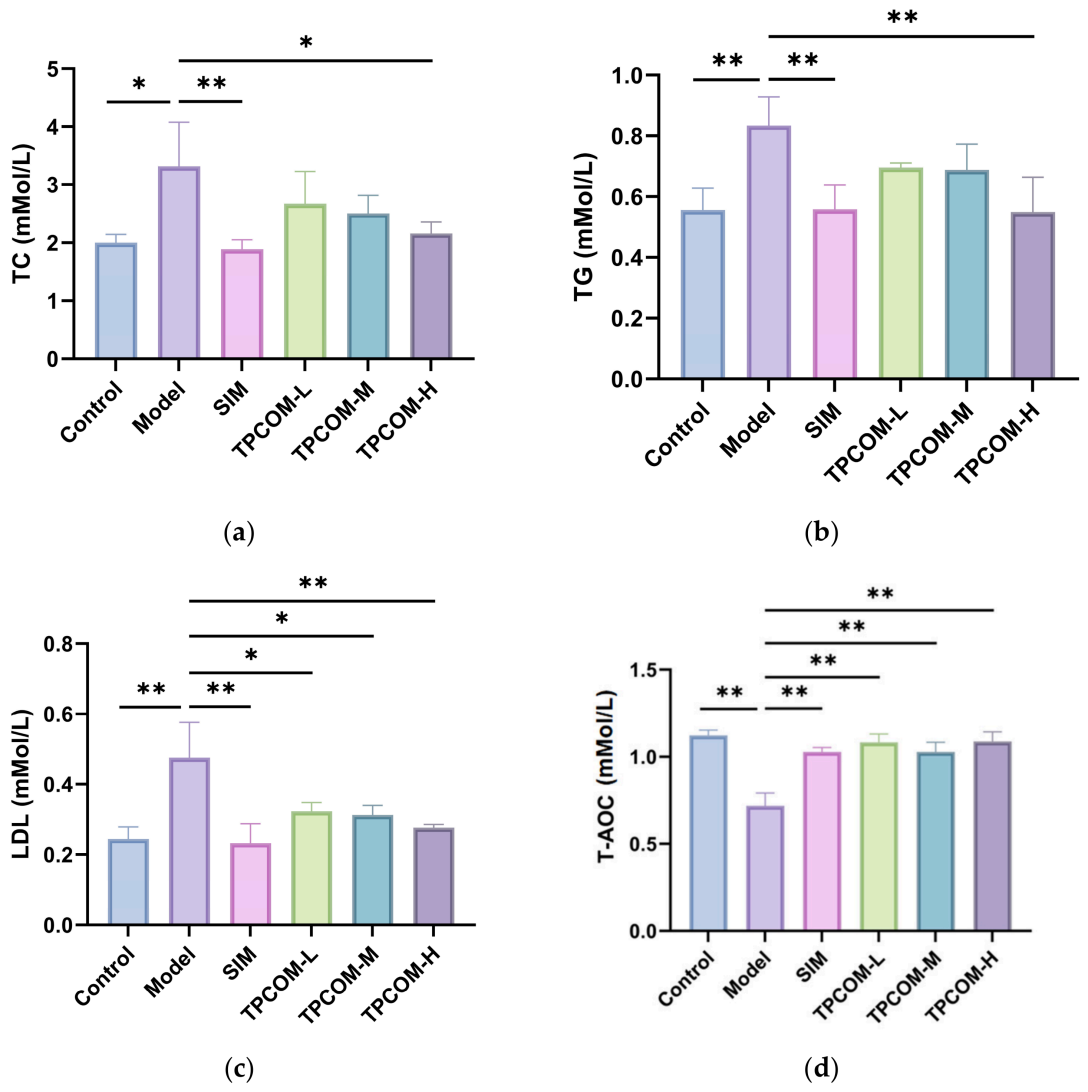
Serum biochemical parameters in mice. (**a**) TC; (**b**) HDL-C; (**c**) LDL-C; (**d**) T-AOC. Data are expressed as mean ± SD. * *p* < 0.05, ** *p* < 0.01 vs. model group.

The results of biochemical analysis in vivo are shown in Figure 8; the levels of serum triglycerides (TG) and total cholesterol (TC) in the HFD group were significantly higher than those in the NC group. Compared with the HFD group, blood lipid levels in the TPCOM intervention groups were significantly improved. The regulatory function of TPCOM was observed from the four blood lipid indicators of serum total triglycerides (TG), low-density lipoprotein cholesterol (LDL-C), total cholesterol (TC), and high-density lipoprotein cholesterol (HDL-C), with significant differences and an improvement trend [15].

### 2.5. TPCOM Alleviated Hepatic Steatosis Verified by H&E Staining

**Figure 9:**
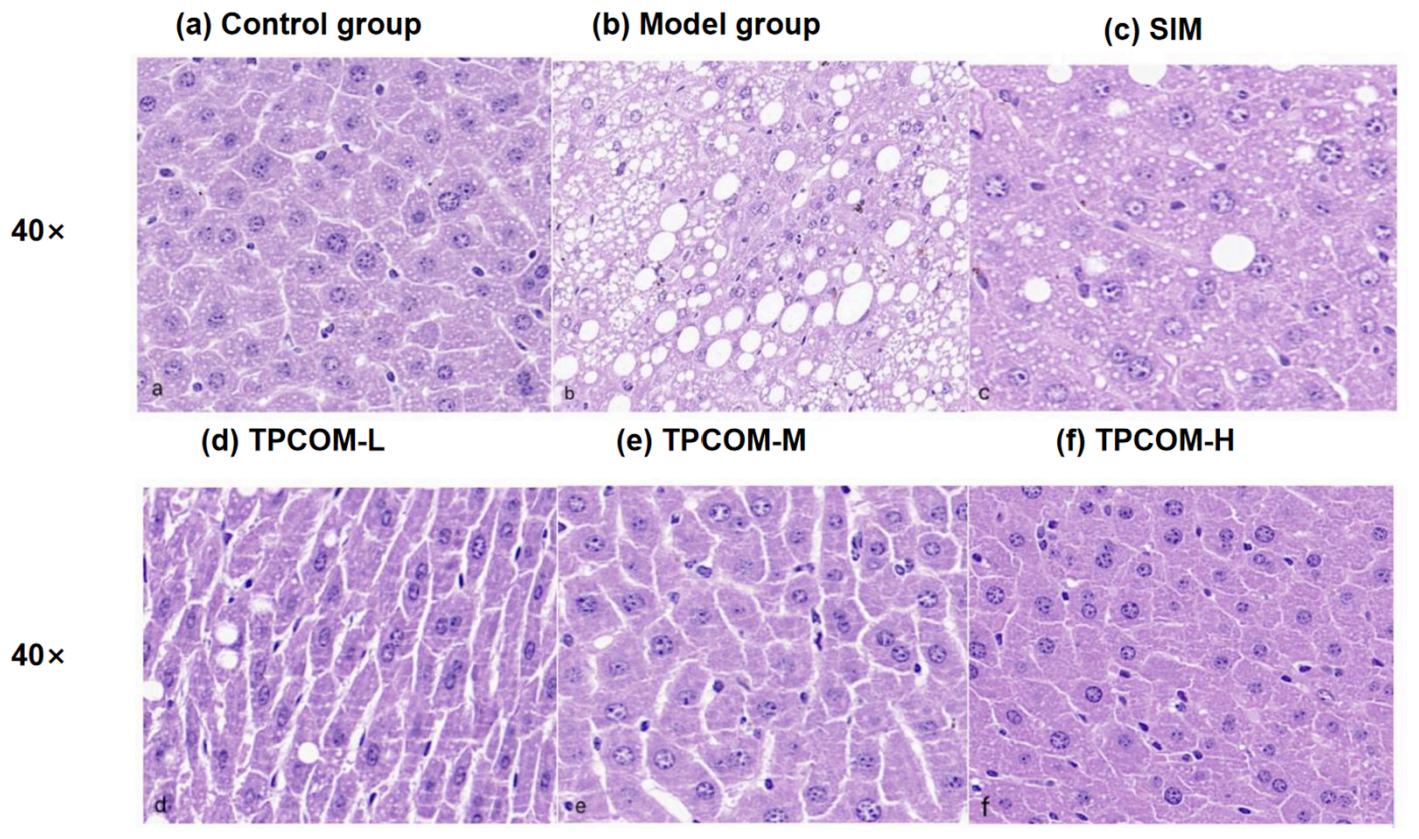
Representative H&E staining micrographs of mouse hepatic tissue, all captured at identical 40× objective magnification. All quantitative data are presented as mean ± standard deviation (SD) with corresponding 95% confidence intervals (95% CI). Statistical analysis was performed via one-way analysis of variance (one-way ANOVA) followed by Tukey’s multiple comparison post hoc test. Significance markers: * *p* < 0.05, ** *p* < 0.01 versus Model group. Exact sample size: n = 10 independent liver tissue samples per experimental group; 5 random non-overlapping microscopic fields were blindly selected for quantitative analysis per sample. Lipid vacuole area fraction was quantified via blinded ImageJ software analysis. Subgraph labels: (**a**) Control group; (**b**) Model group; (**c**) SIM positive control group; (**d**) low-dose TPCOM (TPCOM-L); (**e**) medium-dose TPCOM (TPCOM-M); (**f**) High-dose TPCOM (TPCOM-H).

Hematoxylin and eosin staining was performed to intuitively observe intrahepatic lipid vacuole accumulation (Figure 9). Hepatocytes of the Control group were intact and regularly arranged, with almost no cytoplasmic lipid droplets. The Model group exhibited extensive large and medium lipid vacuoles inside hepatocytes, forming typical macrovesicular fatty liver lesions, consistent with elevated liver weight and abnormal serum lipid indicators. TPCOM intervention relieved hepatic lipid deposition in a dose-dependent trend: TPCOM-L slightly reduced lipid droplet area; TPCOM-M markedly shrank intracellular fat vacuoles and recovered hepatic tissue structure; TPCOM-H nearly restored liver lipid accumulation to normal levels, with histopathological improvement equivalent to the SIM group. These morphological results confirmed that TPCOM can effectively inhibit ectopic lipid deposition in the liver and mitigate HFD-induced non-alcoholic fatty liver injury [16].

Quantitative analysis of hepatic lipid area fraction was performed with blinded image processing and standardized ImageJ calculation (n = 10 liver samples per group, 5 random non-overlapping 40× fields per sample). The average lipid area percentage of the Model group was significantly higher than that of the Control group (## *p* < 0.01). After gradient TPCOM intervention, the hepatic lipid area fraction decreased in a dose-dependent manner: TPCOM-L produced a mild reduction (* *p* < 0.05), while TPCOM-M and TPCOM-H significantly lowered lipid accumulation (** *p* < 0.01). All representative micrographs presented in Figure 9 were acquired at identical 40× magnification for fair visual comparison.

### 2.6. TPCOM Upregulated Glycolipid Metabolism-Related Proteins in Hepatic Tissue

**Figure 10:**
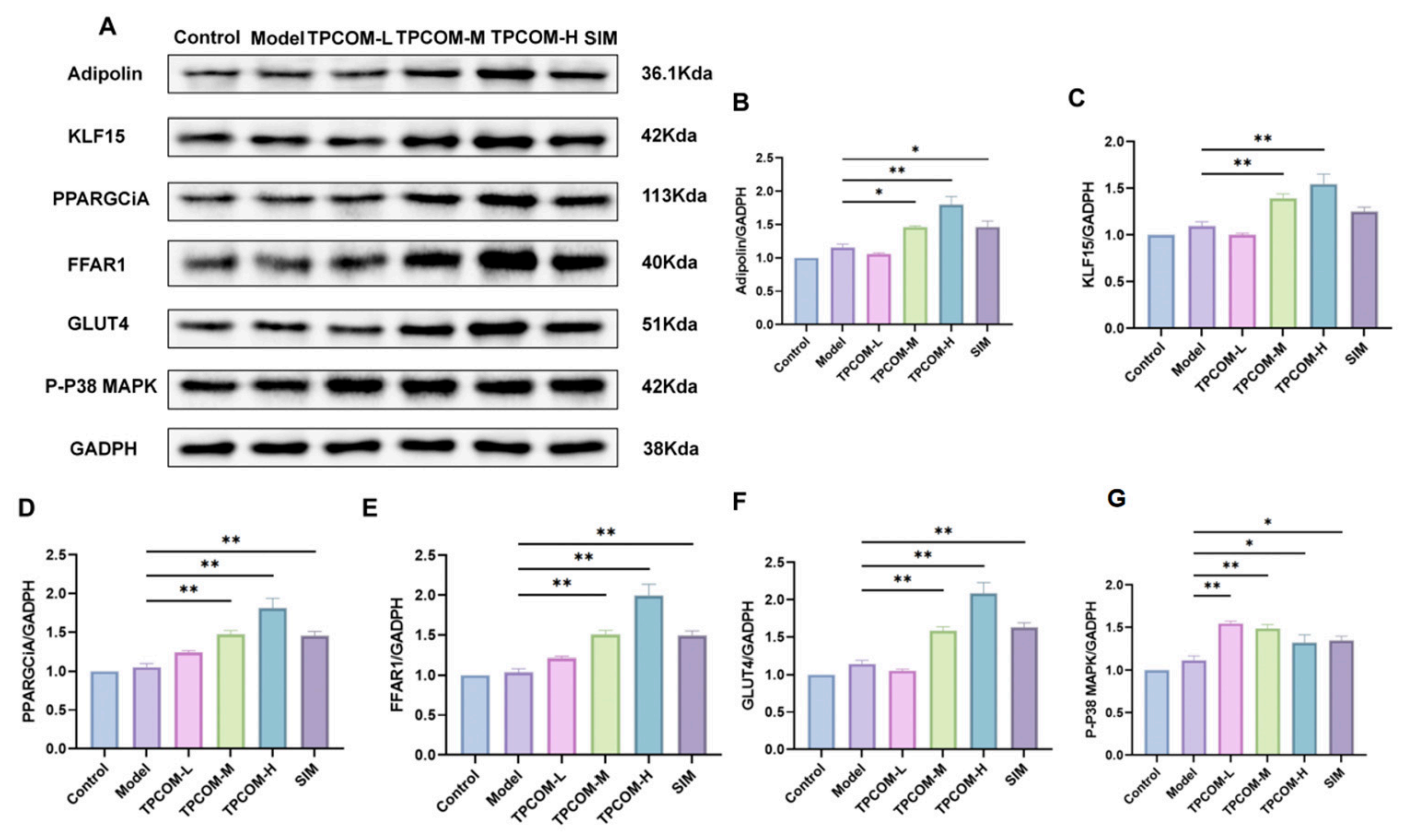
Western blot bands and quantitative relative expression of glycolipid metabolism-related proteins in liver (n = 10 biological replicates per group). (**A**) Original protein bands; (**B**–**G**) statistical histograms normalized to GAPDH. * *p* < 0.05, ** *p* < 0.01 vs. Model group.

Western blot was used to detect six core proteins (Adipolin, KLF15, PPARGC1A, FFAR1, GLUT4, phosphorylated p38 MAPK) screened by network pharmacology, with GAPDH as an internal reference for gray value normalization (Figure 10, n = 10 liver biological replicates per group). Compared with the Control group, the relative expression levels of Adipolin, KLF15, PPARGC1A, FFAR1, GLUT4 and p-p38 MAPK were significantly downregulated in Model mice (## *p* < 0.01), while total p38 MAPK protein expression showed no obvious inter-group difference. After TPCOM administration, the expression of these target proteins increased gradually with dosage, and the TPCOM-H group exhibited the highest protein abundance with significant statistical difference versus Model (* *p* < 0.05 or ** *p* < 0.01).

These six proteins participate in mitochondrial biogenesis, insulin-dependent glucose transport, fatty acid oxidation and lipid-sensing signal transduction. The dose-dependent upregulation of these hub proteins implied that TPCOM may regulate hepatic glycolipid homeostasis via the hypothesized FFAR1–PPARG–p38 MAPK signaling cascade. It is critical to clarify that only correlative changes of protein expression were observed in this research. Gene silencing or specific pathway inhibitor experiments are still required to further verify the direct causal relationship and upstream–downstream regulatory order within this protein axis.

Notably, the significant upregulation of Adipolin, KLF15, PPARGC1A, FFAR1, GLUT4 and phosphorylated p38 MAPK only reflects correlative changes in hepatic protein abundance after TPCOM intervention. The current study lacks pathway inhibition, gene silencing or knockout experiments, so we cannot confirm that the altered expression of these proteins directly causes the anti-obesity, lipid-lowering and hepatoprotective effects of TPCOM. The FFAR1–PPARG–p38 MAPK signaling cascade summarized herein is a preliminary hypothesis integrated from network pharmacology prediction and in vivo protein detection, and direct causal verification is still needed in subsequent functional studies [17].

## 3. Discussion

Different from our prior phytochemical research that merely characterized the chemical composition of *Cydonia oblonga Miller* fruit polyphenols, the novelty of the present work lies in the systematic in vivo pharmacological and preliminary mechanistic exploration of TPCOM’s anti-obesity activity. The UPLC-MS/MS polyphenol quantification data (Table 1 and Table 2) cited herein are reproduced from our previously published study only to define the chemical profile of the raw material used for animal treatment; all phenotypic, biochemical, histopathological and molecular protein data reported in this manuscript represent original experimental outputs generated independently in the current research. To our knowledge, this study is the first to correlate the abundant characteristic polyphenols in Xinjiang quince with a tentative FFAR1–PPARG–p38 MAPK protein cascade via combined bioinformatic prediction and hepatic protein detection, and provides correlative in vivo molecular clues to interpret how TPCOM alleviates HFD-triggered metabolic dysfunction. This work delivers a clear extension of our earlier single-component chemical analysis and generates new preclinical evidence supporting the metabolic regulatory potential of COM fruit [18].

Obesity has developed into a worldwide public health crisis accompanied by chronic low-grade inflammation, ectopic lipid deposition, insulin resistance, and elevated susceptibility to type 2 diabetes and non-alcoholic fatty liver disease (NAFLD), as well as cardiovascular complications. Standard lifestyle modification is the primary intervention strategy, yet long-term compliance remains poor, while synthetic anti-obesity drugs are often restricted by adverse side effects, drug tolerance and high treatment costs. Plant-derived polyphenols have gained increasing attention as candidate metabolic regulators owing to their biosafety and multi-homeostatic regulatory properties, rather than single-pathway inhibitory effects. In this work, we integrated UPLC-MS/MS phytochemical identification, in silico network pharmacology, in vivo mouse phenotypic observation, serum biochemistry, hepatic histopathology and Western blot detection to systematically evaluate the anti-obesity and glycolipid-modulating capacity of TPCOM from Xinjiang medicinal and edible quince. Our convergent experimental data demonstrate that TPCOM can dose-dependently relieve HFD-induced obesity, hyperlipidaemia and hepatic lipid accumulation, with correlative hepatic protein changes pointing to a tentative FFAR1–PPARG–p38 MAPK-related regulatory network [19].

UPLC-MS/MS profiling verified that purified TPCOM contains abundant structurally diverse polyphenol monomers, with chlorogenic acid, cryptochlorogenic acid, quercetin 3-rutinoside and kaempferol glycosides as the dominant bioactive constituents. Existing independent cell and animal studies have documented that each of these phenolic compounds exerts modulatory effects on adipogenesis, lipid catabolism and glucose homeostasis. Chlorogenic acid suppresses early differentiation of preadipocytes and accelerates hepatic fatty acid oxidation via AMPK-dependent pathways to inhibit de novo lipogenesis. Quercetin and its glycoside derivatives ameliorate insulin responsiveness, facilitate plasma membrane translocation of GLUT4, mitigate inflammatory cascades and boost mitochondrial biogenesis in cell models. Kaempferol glycosides have also been reported to suppress lipid synthesis and promote energy expenditure. These polyphenolic components do not function in a simple additive manner; instead, they act synergistically by targeting overlapping and complementary nodes across metabolic signalling networks, which may explain why crude polyphenol extracts frequently exert more balanced, sustained metabolic benefits than single purified monomers. Nevertheless, the current study cannot quantify the individual contribution of each isolated polyphenol or clarify their interactive synergistic effects within the complex extract mixture, which constitutes a key direction for follow-up monomer separation and independent functional verification [20].

Network pharmacology and molecular docking analyses were only applied as hypothesis-generating in silico tools in this research, rather than definitive functional proof. After intersecting TPCOM-derived targets and obesity-associated genes, GO and KEGG enrichment analyses highlighted PPARG, FFAR1 and p38 MAPK as hub molecules enriched in lipid and glucose metabolic pathways, laying a preliminary predictive foundation for subsequent protein detection. Western blot measurement of hepatic tissue only captured parallel correlative shifts in the expression of FFAR1, PPARγ, phosphorylated p38 MAPK, PPARGC1A and GLUT4 upon TPCOM administration. Based on the integrated computational and in vivo correlative data, we proposed a hypothesized FFAR1–PPARG–p38 MAPK protein cascade that may be involved in TPCOM-mediated metabolic improvement. It is critical to emphasise that the enrichment results from bioinformatics merely offer directional predictive clues, and altered protein abundance in liver tissue only reflects simultaneous correlation without establishing direct causal relationships [21].

The parallel upregulation of FFAR1, PPARγ and phosphorylated p38 MAPK in TPCOM-treated mouse livers exhibited consistency with the top-ranked PPAR and MAPK signalling pathways identified via enrichment analysis, which tentatively implies that this group of lipid-sensing proteins may be associated with the alleviation of HFD-induced dyslipidemia and hepatic steatosis. However, without gene silencing, knockout animal models or specific pathway inhibitor intervention experiments, we are unable to confirm the exact upstream-to-downstream hierarchical activation order among these target proteins. Further cell-level and gene-modified animal assays are required to validate whether this protein cascade exerts indispensable metabolic regulatory functions [22].

From a speculative mechanistic perspective supported only by correlative protein data, the co-expressed hub proteins detected in liver tissue may form a coordinated regulatory module centred on PPARG–FFAR1–p38 MAPK. PPARG is a well-documented transcription factor governing adipocyte differentiation and lipid storage, while FFAR1 acts as a membrane lipid sensor to modulate insulin secretion and peripheral glucose uptake. The concurrent elevation of both proteins in TPCOM-treated samples may reflect reciprocal functional crosstalk contributing to metabolic balance, though this interaction remains unvalidated via direct functional tests. The potential downstream effector PPARGC1A serves as a master transcriptional co-activator linked to mitochondrial biogenesis and oxidative metabolism, whereas GLUT4 is the rate-limiting transporter responsible for insulin-dependent glucose clearance. Notably, p38 MAPK phosphorylation was selectively increased without significant variation in total p38 MAPK protein levels. Although p38 MAPK is widely recognised as a stress-responsive inflammatory kinase, moderate activation of phosphorylated p38 MAPK has been reported to facilitate fatty acid oxidation and glucose utilisation under non-pathological mild stimulation. In our HFD mouse model, the elevated p-p38 MAPK level displayed a positive correlation with PPARGC1A, FFAR1 and GLUT4 abundance, which tentatively suggests a metabolically favourable activation pattern distinct from pro-inflammatory p38 signalling. All the above mechanistic interpretations remain tentative speculations rather than confirmed causal pathways, as we lack targeted interference experiments to verify whether TPCOM directly triggers sequential activation of this protein axis to redirect metabolic flux toward lipid breakdown and glucose consumption [23].

We stress that the FFAR1–PPARG–p38 MAPK cascade proposed herein is derived from multi-layer correlative evidence including in silico prediction, animal phenotypic data, histological observation and hepatic protein profiling, yet no direct causal validation experiments were conducted in the present study. Genetic knockout, siRNA interference or pharmacological blocking assays are required to establish definitive functional connections between these hub proteins and TPCOM’s anti-obesity phenotype. Accordingly, this protein regulatory network only represents a biologically plausible mechanistic hypothesis, rather than a fully validated signalling pathway [24].

At the physiological phenotypic level, TPCOM intervention produced dose-dependent mitigation of HFD-induced weight gain, hyperlipidaemia and hepatic lipid deposition, which constitute robust, directly measurable experimental outcomes. Minor inconsistent trends were observed for several serum lipid markers within the low-dose TPCOM group, a common phenomenon in multi-component natural product research. Such slight fluctuations can be attributed to concentration threshold effects, biological individual variation and differential tissue sensitivity to phytochemical regulation [25,26]. Body weight serves as a comprehensive integrated marker of whole-body energy balance and responds sensitively to interventions that boost energy expenditure, while circulating lipid levels are jointly controlled by hepatic lipogenesis, intestinal lipid absorption and peripheral tissue uptake, alongside circadian metabolic oscillations, which may generate less linear dose-response trends. These subtle discrepancies do not negate the overall anti-obesity efficacy of TPCOM; instead, they reflect the multi-target, gradual homeostatic modulation characteristic of botanical polyphenols [27,28].

TPCOM treatment also markedly elevated serum total antioxidant capacity (T-AOC) via two plausible indirect mechanisms. On one hand, abundant phenolic hydroxyl groups within TPCOM polyphenols can directly neutralise reactive oxygen species and upregulate endogenous antioxidant enzyme activity. On the other hand, the alleviation of visceral fat accumulation secondary to TPCOM intervention relieves chronic systemic oxidative stress, which in turn improves circulating antioxidant status. In obese states, excessive oxidative stress impairs insulin signal transduction and accelerates intrahepatic lipid deposition; the elevated T-AOC observed in this study can be regarded as a correlative marker of recovered metabolic homeostasis, yet independent antioxidant functional tests are required to confirm causal antioxidant effects of TPCOM [29].

A critical interpretative limitation of the current work is that all detected shifts in hepatic glycolipid regulatory protein expression represent bidirectional correlative changes rather than unidirectional causal signals. TPCOM polyphenols may directly bind and modulate the transcription or translation of FFAR1, PPARGC1A, GLUT4 and p38 MAPK to drive metabolic improvement; simultaneously, the relief of obesity-induced lipid overload and oxidative stress may trigger adaptive compensatory upregulation of these homeostatic proteins. We cannot distinguish the primary causal regulatory events from secondary adaptive protein alterations based solely on static post-intervention protein expression data, which highlights the complexity of systemic metabolic networks and restricts single-target mechanistic explanations [30].

Several unavoidable limitations of this study must be acknowledged to avoid overinterpretation of the tentative mechanistic model. First, the individual metabolic function and synergistic interactions of each isolated polyphenol monomer within TPCOM remain uncharacterized. Second, direct functional validation experiments (gene silencing, knockout mice, pathway inhibitors) are absent, so the causal hierarchical relationships within the hypothesized FFAR1–PPARG–p38 MAPK cascade cannot be confirmed. Third, all experimental observations are limited to HFD-induced male mouse models, and human clinical trials are still required to evaluate oral bioavailability, in vivo safety and translational therapeutic value of TPCOM [31].

In summary, consistent phenotypic, serum biochemical and histopathological evidence collectively demonstrates that TPCOM exerts dose-dependent anti-obesity, lipid-regulating and hepatoprotective effects on HFD-fed mice. Correlative hepatic protein expression shifts combined with in silico bioinformatic prediction generate a tentative multi-component, multi-target regulatory hypothesis centred on the FFAR1–PPARG–p38 MAPK protein cascade, which may be associated with enhanced mitochondrial homeostasis, insulin-related glucose uptake, fatty acid oxidation and systemic antioxidant defence, alongside reduced hepatic steatosis and obesity-associated oxidative stress. Nevertheless, all pathway-related interpretations in this work remain unvalidated speculative mechanisms limited by the lack of gene and pharmacological interference assays. Integrated UPLC-MS/MS chemical characterisation, computational prediction and in vivo preclinical data provide preliminary modern pharmacological evidence supporting the traditional medicinal value of Xinjiang quince fruit, and lay a foundational reference for further research into TPCOM as a candidate natural functional food or auxiliary agent for obesity and metabolic syndrome management [32].

## 4. Materials and Methods

### 4.1. Preparation and Chemical Characterization of TPCOM

COM were collected from Kashgar Prefecture, Xinjiang, China, and authenticated by Prof. Palida Abulizi (Xinjiang Uygur Autonomous Region Institute of Pharmaceutical Research, Urumqi, China). The polyphenol-rich extract of COM fruit (TPCOM) was prepared as previously described. Briefly, the crude extract was obtained from quince fruit and subsequently purified using AB-8 macroporous resin to enrich the polyphenolic fraction. The yield of the unpurified extract was 25.54%, whereas the yield of the purified extract was 4.26%.

The extract used in the present study was prepared using the same extraction and purification procedure, and the previously published phytochemical data were used as the basis for its chemical characterization and quality control. The extract used in the present study was from the same preparation batch as that characterized previously.

### 4.2. Network Pharmacology

#### 4.2.1. Screening for TPCOM and Obesity Targets

We gathered and anticipated targets of TPCOM utilizing the PubChem Database and TCMSP Platform (Traditional Chinese Medicine Systems Pharmacology Database and Analysis Platform). TCMs are frequently taken orally when used in clinical treatment. ADME-related models, including drug-likeness (DL) and oral bioavailability (OB) [33], primarily impact drug absorption via the GIT. Following criteria like OB ≥ 20% and DL ≥ 0.18, these thresholds (OB ≥ 20% and DL ≥ 0.18) are widely accepted and commonly used criteria in the TCMSP database and network pharmacology studies for natural products, as they effectively enrich bioactive components with favorable oral bioavailability and drug-likeness. Relevant references have been added to support these criteria. The bioactive components were further characterized, and the associated targets for each component were identified. The next step was to search for disease targets utilizing the term “Obesity” in the following databases: OMIM Database (an Online Catalog of Human Genes and Genetic Disorders), GeneCards Database (The Human Gene Database), and DrugBank Database under the standards of Score ≥ 20. The GeneCards database (Version 5.22) was used to search for disease targets with obesity (Obesity) as the keyword, and Relevance.score was set greater than 5.

#### 4.2.2. Network Construction

The compound–target interaction network was developed utilizing Cytoscape software, particularly its version 3.9.1. This specific version facilitated the smooth incorporation of active compounds and their corresponding targets pertinent to the drug of interest. Subsequently, we obtained PPIs through the extensive utilization of the STRING database resources. Then, leveraging the PPIs obtained, we once again utilized Cytoscape version 3.9.1 to construct a meticulous protein–protein interaction network. This network provided a thorough visual depiction of the complex interactions among the proteins, enabling a detailed analysis and interpretation of the data.

#### 4.2.3. Functional Enrichment Analysis

Gene Ontology analysis consists of three fundamental aspects: Biological Processes (BPs), Molecular Functions (MFs), and Cellular Components (CCs). These components collectively aim to elucidate the functional attributes of genes [34]. To better characterize the hub genes discovered in our study, we utilized the Kyoto Encyclopedia of Genes and Genomes (KEGG) for enrichment analysis. This helped us to identify and explore the enrichment of diverse biological signaling pathways. We subsequently applied the R programming language 4.3.0 and the Bioconductor ClusterProfiler package 4.4.4 for the analyses of gene ontology and KEGG enrichment. Our integration of the results from our first analyses within the R environment and execution of the ClusterProfiler package enabled us to perform enrichment analysis based on a gene-cluster-specific level for a more microscopic look. This procedure was intended to provide insight into the functional characteristics and interaction patterning of those identified hub genes for a more global appreciation of their roles [35].

#### 4.2.4. Molecular Docking

Target Protein and Ligand Preparation:Based on the intersection targets of TPCOM and obesity obtained from network pharmacology, combined with GO/KEGG enrichment results and reported metabolic regulatory functions, five core hub proteins related to glycolipid homeostasis were selected for molecular docking analysis: PPARG, FFAR1, p38 MAPK, PPARGC1A, and GLUT4. The 3D crystal structures of target proteins were downloaded from the RCSB PDB database with unique PDB IDs as follows: PPARG (PDB: 3DZY), FFAR1 (PDB: 4PHU), p38 MAPK (PDB: 1LEW), PPARGC1A (PDB: 5V0W), GLUT4 (PDB: 6THA). All protein crystal structures were processed via PyMOL 2.5 software: water molecules, irrelevant heteroatoms and original co-crystallized ligands were removed; polar hydrogens were added, and partial atomic charges were assigned with the Gasteiger algorithm. The processed protein files were saved in PDBQT format for subsequent docking.

The 10 dominant polyphenol ligands identified by UPLC-MS/MS (neochlorogenic acid, chlorogenic acid, cryptochlorogenic acid, caffeic acid, quercetin 3-rutinoside, quercetin 3-galactoside, quercetin 3-glucoside, kaempferol 3-rutinoside, kaempferol 3-glucoside, isochlorogenic acid) were retrieved as 2D SDF structures from the PubChem database. Ligand structures were optimized with the MMFF94 force field, geometric minimization was conducted to reach a gradient convergence threshold of 0.01 kcal/(mol·Å), polar hydrogens and Gasteiger charges were added, and ligands were exported as PDBQT files.

Docking Software and Parameter Setting:AutoDock Vina 1.2.3 was adopted as the molecular docking calculation software. A semi-flexible docking protocol was applied: all ligand chemical bonds were set rotatable, while target protein backbones and side chains were kept rigid. For each protein’s active binding pocket, the 3D docking box center coordinates (x, y, z) and box size (width × height × depth, unit: Å) were determined according to the position of the original co-crystallized ligand in the crystal structure; detailed box parameters are listed in Table 4.

Docking Method Validation:Self-docking (redocking) validation was performed to evaluate the reliability of our docking workflow. The native co-crystallized ligand of each protein crystal was extracted, preprocessed with the same ligand preparation pipeline, and docked back into the corresponding protein pocket using identical box and Vina parameters. The root-mean-square deviation (RMSD) value between the re-docked optimal conformation and the original crystal ligand conformation was calculated via PyMOL. All RMSD values of five target proteins were <2.0 Å (PPARG: 1.16 Å; FFAR1: 1.32 Å; p38 MAPK: 0.98 Å; PPARGC1A: 1.45 Å; GLUT4: 1.21 Å). RMSD < 2 Å is widely recognized as the standard for reliable docking simulation, confirming that our docking parameter system can accurately reproduce natural ligand–protein binding modes.

Post-Docking Analysis and Restrictive Interpretation:After calculation, the binding conformation with the lowest binding free energy (most negative value) was selected as the optimal binding mode for each ligand–target pair. Hydrogen bonds, hydrophobic interactions and binding pocket residues were visualized and analyzed by PyMOL 2.5. Binding free energy ranges of main polyphenol–target complexes were −7.2~−9.6 kcal/mol.

Critical interpretative limitation: It must be emphasized that molecular docking only simulates static molecular binding affinity under in silico conditions, which cannot reflect dynamic intracellular environments, compound metabolic transformation, or actual signal transduction activation in living organisms. Docking results merely provide preliminary predictive clues for potential ligand–target interactions, and cannot independently confirm biological functional effects of TPCOM. All docking predictions need to be supported by subsequent in vivo protein detection experiments.

### 4.3. Animals and Treatments

Six-week-old male SPF-grade C57BL/6J mice with initial body weight of 26 ± 2 g (total 60 individuals) were purchased from qualified laboratory animal suppliers. The whole animal experimental scheme was approved by the Experimental Animal Ethics Committee of Xinjiang Medical University (Approval No. IACUC-20190226). All animal operations, modeling, drug intervention and tissue collection strictly complied with the ARRIVE 2.0 guidelines and the NIH Guide for the Care and Use of Laboratory Animals. Mice were housed in SPF-level barrier facilities with a 12 h light–dark cycle, constant ambient temperature of 22–24 °C and relative humidity maintained at 40–45%. Sterilized standard chow and autoclaved purified water were provided ad libitum; corn cob bedding was replaced 2–3 times per week to keep cages hygienic. All mice received a 7-day adaptive acclimation before formal modeling experiments.

#### 4.3.1. Randomization Procedure

After 7 days of adaptive feeding, all mice were weighed and labeled with unique ear tags. Random number matrices were generated using GraphPad Prism 9.0 software to realize stratified random grouping for balancing baseline body weight. Initially, 10 mice were assigned to the normal control group fed standard chow, while the remaining 50 mice received a high-fat diet (HFD, D12492, 60 kcal% fat) for 12 weeks to establish obesity models. After successful modeling, the 50 obese mice were further randomly divided into five equal subgroups via a second random number table, with 10 mice per subgroup. To eliminate cage-induced bias, mice were randomly rearranged into different cages every two weeks during modeling and drug administration periods. The final six experimental groups all contained 10 mice per cohort.

#### 4.3.2. Blinding Implementation

A full single-blind design was adopted throughout the whole experimental workflow. All researchers responsible for oral gavage, weekly body weight recording, food intake statistics, tissue dissection, paraffin embedding, H&E slice scanning, serum biochemical testing and Western blot gray quantification were kept blind to group information. Group coding was only decoded after all raw data collection and quantitative analysis were fully finished to avoid artificial subjective measurement bias.

#### 4.3.3. Sample Size Justification

A uniform sample size of n = 10 mice per group was set for all phenotypic, biochemical, histological and molecular detections, with detailed rationale as follows:

In vivo phenotypic indicators (body weight, visceral fat, heart, kidney and liver wet weight): n = 10 mice per group can guarantee sufficient statistical power (α = 0.05) to identify inter-group weight differences, consistent with numerous published HFD-induced obesity mouse pharmacology studies.

Ex vivo detections (serum lipid and T-AOC assay, hepatic H&E lipid quantitative analysis, hepatic Western blot): All tests adopted independent biological samples taken from every single mouse, meaning n = 10 biological replicates for each index.

#### 4.3.4. Grouping and Drug Administration

Six experimental groups (n = 10 per group) were established: Control group: Standard chow + equal volume 0.9% normal saline (10 mL/kg, oral gavage daily); Model group: HFD + equal volume 0.9% normal saline; SIM positive control group: HFD + simvastatin 5 mg/kg/d; TPCOM-L low-dose group: HFD + TPCOM 125 mg/kg/d; TPCOM-M medium-dose group: HFD + TPCOM 200 mg/kg/d; TPCOM-H high-dose group: HFD + TPCOM 250 mg/kg/d.

TPCOM powder and simvastatin tablets were dissolved in sterile 0.9% normal saline and prepared fresh every day. The three gradient TPCOM doses were determined based on our previous 3T3-L1 preadipocyte cytotoxicity screening results; the simvastatin dosage was selected referring to widely used lipid-regulating studies on obese mice [36]. All treatments were given via daily oral gavage for 6 consecutive weeks. Body weight and food consumption were recorded weekly, and animal mental state, fur loss and mortality were observed daily.

#### 4.3.5. Tissue and Plasma Sample Collection

After the last oral gavage, all mice were fasted for 8 h with free access to water, then anesthetized via subcutaneous injection of pentobarbital sodium at a dose of 35 mg/kg. Orbital venous blood was collected with heparinized tubes and stood at room temperature for 40 min, followed by centrifugation at 3000 r/min and 4 °C for 15 min to isolate plasma. Plasma samples were sub-packed and stored at −80 °C for serum biochemical detection (n = 10 plasma samples per group). Visceral fat, heart, kidney and liver tissues were quickly dissected and weighed immediately. Each liver tissue was divided into two parts: one part was fixed in 4% paraformaldehyde for 24 h for H&E staining (n = 10 liver samples per group); the other was snap-frozen in liquid nitrogen and transferred to −80 °C refrigerator for total protein extraction and Western blot detection (n = 10 liver biological replicates per group).

#### 4.3.6. Statistical Pre-Assumptions

All datasets underwent two prerequisite tests before parametric statistical analysis: Shapiro–Wilk normality test and Levene homogeneity of variance test. All body weight, organ weight, serum biochemistry and protein quantification data conformed to normal distribution (*p* > 0.05) and exhibited equal variance across groups (*p* > 0.05). Therefore, one-way ANOVA combined with Tukey’s post hoc multiple comparison test was adopted for inter-group difference analysis, and nonparametric tests were not required in this study.

### 4.4. Serum Analysis

Blood samples were collected in 1.5 mL enzyme-free Eppendorf tubes. After allowing the blood to clot at room temperature for 40 min, it was centrifuged at 3000 rpm for 10 min to separate the serum. Serum lipid profiles, including total cholesterol (TC), triglycerides (TG), low-density lipoprotein-cholesterol (LDL-C), and high-density lipoprotein-cholesterol (HDL-C), were assessed using enzymatic kits (A110-1-1, A111-1-1, A112-1-1, and A113-1-1, Nanjing Jiancheng Bioengineering Institute, Nanjing, China). All measurements were performed according to the manufacturer’s instructions.

### 4.5. Hematoxylin and Eosin Staining

For hematoxylin and eosin (H&E) staining, both liver and adipose tissues were immersed in 4% PFA and kept at 4 °C for overnight fixation. The tissues were embedded in paraffin, and sections of 4 μm in thickness were subsequently prepared. Each section was sequentially processed for dewaxing and hydration (xylene, 100% ethanol, 80% ethanol, 70% ethanol, and ddH_2_O). The nuclei were stained with hematoxylin for 4 min. Then, each section was rinsed in tap water for 10 min and stained with eosin for 10 s. Finally, each section was sequentially placed in 70% ethanol, 80% ethanol, 95% ethanol, and 100% ethanol for gradient dehydration, as well as xylene and butanol for transparency.

After paraffin sectioning and H&E staining, all hepatic tissue slides were scanned with a digital pathological scanner. For quantitative analysis of intrahepatic lipid vacuole area. Each section was examined with an intelligent digital slicing scanner (EasyScan, Motic, Xiamen, China) at 40× magnifications. The adipocyte size was quantified using ImageJ software (NIH, Bethesda, MD, USA) on 40× magnification images.

Blinding operation: All slice labeling was concealed with random digital codes before scanning and image analysis; the operator was completely blinded to group information during field selection and lipid area quantification, and group codes were unlocked only after all quantitative statistics were completed.

Field selection standard: For each liver sample (n = 10 per group), 5 non-overlapping, randomly selected 40× magnification visual fields were captured manually, avoiding the central vein and large vascular regions to eliminate sampling bias.

ImageJ quantification workflow: All captured images were imported into ImageJ v1.54f software. Images were converted to 8-bit grayscale, uniform threshold values were set to separate lipid vacuole white empty regions from hepatocyte parenchyma; the total area of lipid droplets in each field was calculated. The lipid area fraction (%) = total lipid vacuole area/total tissue area of this field × 100. The average lipid area percentage of 5 random fields from one slice was taken as the quantitative value of a single mouse liver sample.

Magnification unification: All representative micrographs displayed in Figure 9 were captured under consistent 40× objective magnification to ensure uniform visual comparison across six experimental groups.

### 4.6. Western Blot Analysis

The frozen tissues were homogenized on ice using a lysis buffer (C1053, Applygen, Beijing, China) containing 50 mM Tris-HCl (pH 7.4), 150 mM NaCl, 1% NP-40, 0.1% SDS, and protease inhibitor (G2007, Servicebio, Wuhan, China). Tissue lysates were centrifuged for 15 min at 12,000 rpm at 4 °C. The supernatant was collected, followed by an assessment of the protein concentration utilizing the BCA Protein Assay Kit (P1511, Applygen, Beijing, China). Then, the supernatant in protein loading buffer was boiled at 95 °C for 10 min. Proteins were separated on 4–12% SDS-PAGE and transferred to a PVDF membrane (IPVH00010, Millipore, Darmstadt, Germany). Blots were incubated with primary antibodies in blocking buffer overnight at 4 °C. The blots underwent three washes with TBST and were then stained with a horseradish peroxidase-conjugated secondary antibody for 1 h at room temperature. Blots were further washed three times with TBST and visualized with an enhanced chemiluminescence kit (ECL, G2014, Servicebio, Wuhan, China). Images were acquired, and the fluorescent density was quantified utilizing a Bioshin ChemiQ 4800 imaging system (Shanghai Oxiang Scientific Instruments Co., Ltd., Shanghai, China). Band densities were quantified using ImageJ software. Target protein expression levels were normalized to GAPDH as an internal control. Data were obtained from ten independent experiments.

### 4.7. Statistical Analyses

All in vivo animal experiments were carried out with n = 10 independent mice per experimental group. All serum biochemical, hepatic histopathological and Western blot detections adopted 10 individual biological replicates derived from separate mice. All quantitative data were presented as mean ± standard deviation (SD). Statistical markers: ## *p* < 0.01 vs. Control group; * *p* < 0.05, ** *p* < 0.01 vs. Model group.

## 5. Conclusions

This study delivers systematic preclinical evidence for the anti-obesity and glycolipid-regulating activity of total polyphenols extracted from *Cydonia oblonga* Miller fruit (TPCOM), representing a clear advancement over our earlier pure phytochemical characterization work. We established a stable AB-8 macroporous resin purification protocol to obtain high-purity TPCOM, and used UPLC-MS/MS to characterize its major phenolic monomers (Table 1 and Table 2; these compositional data are reproduced from our prior published research solely for raw material identification). All other experimental outputs, including network pharmacology prediction, molecular docking, HFD mouse intervention, serum biochemical detection, quantitative hepatic histopathology and hepatic protein Western blot analysis, are original data generated in the present work [36].

Multi-layer convergent results demonstrate that TPCOM alleviates HFD-induced weight gain, dyslipidaemia, hepatic steatosis and systemic oxidative stress in mice in a dose-dependent manner. Combined in silico prediction and in vivo protein detection provide tentative correlative clues that TPCOM may exert metabolic protection via coordinated regulation of PPARG, FFAR1, p-p38 MAPK, PPARGC1A and GLUT4. This work fills the research gap between the chemical composition of Xinjiang quince polyphenols and their metabolic regulatory function, and provides experimental support for developing TPCOM as a natural functional raw material to intervene in obesity and related metabolic disorders [37].

## 6. Limitations

Although we combined network pharmacology, molecular docking and in vivo Western blot to screen a set of candidate glycolipid regulatory proteins and the hypothesized FFAR1–PPARG–p38 MAPK cascade, the mechanistic evidence of this study remains insufficient to confirm direct causal effects. Only protein expression correlation data from liver tissue were obtained, without targeted functional interference experiments. We did not apply specific pharmacological inhibitors of FFAR1 or p38 MAPK, nor did we perform siRNA gene silencing in adipocyte/hepatocyte models or gene knockout animal models. Without these intervention tools, we cannot distinguish whether the elevated levels of FFAR1, PPARGC1A and p-p38 MAPK are the direct trigger for TPCOM’s metabolic improvement, or just secondary adaptive changes relieved by reduced lipid accumulation and oxidative stress.

Another limitation of the current study is the absence of food efficiency ratio (FER) analysis. Complete cumulative food consumption raw data for individual animals were not fully archived, so retrospective calculation of FER (total body weight gain per unit food intake) could not be performed. Although we confirmed that the average total food intake did not differ significantly across groups, we cannot quantitatively exclude subtle changes in feeding efficiency that may affect body weight gain. Subsequent related animal experiments will fully record individual food consumption and calculate FER to comprehensively clarify whether the anti-obesity activity of TPCOM involves regulation of feeding efficiency.

To resolve this limitation and fully validate the proposed signaling axis, our follow-up research will carry out a series of multi-level functional experiments as follows: First, in vitro cell assays using siRNA-mediated knockdown of core hub genes in 3T3-L1 adipocytes and primary hepatocytes will be performed to test whether TPCOM loses its lipid-regulating activity after target gene silencing. Second, pharmacological blocking experiments with specific FFAR1 and p38 MAPK inhibitors will be conducted on HFD obese mice to observe the reversal of TPCOM’s therapeutic phenotype. Third, FFAR or PPARG knockout mouse models will be adopted to identify the essential function of this protein axis in TPCOM’s anti-obesity action. In addition, we will isolate individual dominant polyphenol monomers from TPCOM to explore their separate and synergistic effects on this candidate pathway.

## Figures and Tables

**Table 1:** UPLC-MS/MS information of the 10 major polyphenolic compounds identified in TPCOM.

No.	Compound	RT (min)	Ion Mode	Precursor Ion (*m*/*z*)	Product Ions (*m*/*z*)	Cone Voltage (V)	Collision Energy (eV)
1	Neochlorogenic acid	2.48	ESI−	353.096	190.93, 134.958	38	18
2	Chlorogenic acid	3.26	ESI−	353.096	190.93, 134.958	38	32
3	Cryptochlorogenic acid	3.39	ESI−	353	191.000, 135.000	40	25
4	Caffeic acid	3.82	ESI−	179	135.000, 107.000	54	20
5	Quercetin 3-rutinoside	4.4	ESI−	609	271.000, 300.000	66	65
6	Quercetin 3-galactoside	5.09	ESI−	463.16	299.988, 270.909	8	28
7	Quercetin 3-glucoside	5.39	ESI−	463	271.000, 300.000	8	36
8	Kaempferol 3-rutinoside	5.96	ESI−	593.149	254.955, 284.885	78	66
9	Kaempferol 3-glucoside	6.32	ESI−	447	255.000, 248.000	58	38
10	Isochlorogenic acid	6.36	ESI−	515	353.000, 191.000	50	15

**Table 2:** Contents of 10 major polyphenolic compounds in purified and unpurified quince fruit extracts.

	Compound	Purified Extract (μg/mg)	Unpurified Extract (μg/mg)
1	Neochlorogenic acid	56.77 ± 1.48	1.16 ± 0.01
2	Chlorogenic acid	160.67 ± 2.51	2.28 ± 0.01
3	Cryptochlorogenic acid	242.44 ± 0.73	3.37 ± 0.01
4	Caffeic acid	1.46 ± 0.05	0.03 ± 0.00
5	Quercetin 3-rutinoside	186.33 ± 1.32	3.13 ± 0.04
6	Quercetin 3-galactoside	40.24 ± 1.46	0.34 ± 0.01
7	Quercetin 3-glucoside	26.72 ± 0.82	0.22 ± 0.00
8	Kaempferol 3-rutinoside	3.10 ± 0.04	0.02 ± 0.00
9	Kaempferol 3-glucoside	1.92 ± 0.06	0.02 ± 0.00
10	Isochlorogenic acid	21.74 ± 0.57	0.05 ± 0.01
	Total	741.41 ± 9.04	10.61 ± 0.09

**Table 3:** Statistics of the number of target points corresponding to the components.

Molecule_Name	MOL_ID	TCMSP_Gene_Num	TCMIP_Gene_Num
Catechol	MOL000089	2	0
Ellagic acid	MOL001002	38	50
Acacetin	MOL001689	25	29
Pectolinarin	MOL005842	12	51
Eupatilin	MOL005734	31	15
Calycosin	MOL000417	22	0
Genkwanin	MOL005573	14	13
Luteolin-4′-0-glucoside	MOL000527	6	9

**Table 4:** Lists docking parameters of five core glycolipid regulatory hub proteins for in vivo verification, whose docking structures are not displayed in Figure 5.

No.	Protein Target	PDB ID	Docking Box Center Coordinates (x, y, z)	Docking Box Size (Width × Height × Depth, Å)
1	PPARG	3DZY	(12.4, 28.7, −5.3)	24 × 24 × 24
2	FFAR1	4PHU	(9.1, −14.2, 3.6)	22 × 22 × 22
3	p38 MAPK	1LEW	(16.8, 22.1, 11.5)	20 × 20 × 20
4	PPARGC1A	5V0W	(−7.3, 19.5, 26.2)	26 × 26 × 26
5	GLUT4	6THA	(32.6, −8.4, 15.7)	22 × 22 × 22

Note: All docking calculations were performed using AutoDock Vina 1.2.3 with semi-flexible docking protocol. The exhaustiveness parameter was set to 32, energy range = 3 kcal/mol, and other parameters were set to default values.

## Data Availability

The original contributions presented in this study are included in the article. Further inquiries can be directed to the corresponding author(s).

## Abbreviations

The following abbreviations are used in this manuscript:

COM	*Cydonia Oblonga Miller*
TPCOM	Total Polyphenol of *Cydonia oblonga Miller*
P38 MAPK	p38 Mitogen-Activated Protein Kinase
GLUT4	Glucose Transporter Type 4
PPARGC1A	PPARG Coactivator 1 Alpha
FFAR1	Free Fatty Acid Receptor 1
KLF15	KLF Transcription Factor 15 Gene
HDL-C	High-Density Lipoprotein Cholesterol
LDL-C	Low-Density Lipoprotein Cholesterol
TC	Total Cholesterol
TG	Triglyceride
T-AOC	Total Antioxidant Capacity
GO	Gene Ontology Analysis
PPI	Protein–Protein Interaction Network
KEGG	Kyoto Encyclopedia of Genes and Genomes
PBS	Phosphate Buffered Saline
ddH_2_O	Doble Distilled H_2_O
mmol/L	Millimoles per Liter
SIM	Simvastatin Tablets
WB	Western Blotting
